# Retreatment with anti-EGFR based therapies in metastatic colorectal cancer: impact of intervening time interval and prior anti-EGFR response

**DOI:** 10.1186/s12885-015-1701-3

**Published:** 2015-10-16

**Authors:** X. Liu, G. C. George, A. M. Tsimberidou, A. Naing, J. J. Wheler, S. Kopetz, S. Fu, S. A. Piha-Paul, C. Eng, G. S. Falchook, F. Janku, C. Garrett, D. Karp, R. Kurzrock, R. Zinner, K. Raghav, V. Subbiah, K. Hess, F. Meric-Bernstam, D. S. Hong, M. J. Overman

**Affiliations:** 1 Department of Investigational Cancer Therapeutics (Phase 1 Clinical Trials Program), The University of Texas MD Anderson Cancer Center, Unit 455, 1515 Holcombe Blvd, Houston, TX 77030 USA; 2 Department of Gastrointestinal Medical Oncology, University of Texas MD Anderson Cancer Center, 1515 Holcombe Boulevard, Unit # 426, Houston, TX 77030 USA; 3 Division of Hematology and Oncology, University of California San Diego Moores Cancer Center, San Diego, CA USA; 4 Biostatistics, University of Texas MD Anderson Cancer Center, Houston, TX USA

**Keywords:** Retreatment, Anti-EGFR treatment, *KRAS*-wt CRC

## Abstract

**Background:**

This retrospective study aims to investigate the activity of retreatment with anti-EGFR-based therapies in order to explore the concept of clonal evolution by evaluating the impact of prior activity and intervening time interval.

**Methods:**

Eighty-nine *KRAS* exon 2-wild-type metastatic colorectal patients were retreated on phase I/II clinical trials containing anti-EGFR therapies after progressing on prior cetuximab or panitumumab. Response on prior anti-EGFR therapy was defined retrospectively per physician-records as response or stable disease ≥6 months. Multivariable statistical methods included a multiple logistic regression model for response, and Cox proportional hazards model for progression-free survival.

**Results:**

Retreatment anti-EGFR agents were cetuximab (n = 76) or cetuximab plus erlotinib (n = 13). The median interval time between prior and retreatment regimens was 4.57 months (range: 0.46-58.7). Patients who responded to the prior cetuximab or panitumumab were more likely to obtain clinical benefit to the retreatment compared to the non-responders in both univariate (p = 0.007) and multivariate analyses (OR: 3.38, 95 % CI: 1.27, 9.31, p = 0.019). The clinical benefit rate on retreatment also showed a marginally significant association with interval time between the two anti-EGFR based therapies (p = 0.053). Median progression-free survival on retreatment was increased in prior responders (4.9 months, 95 % CI: 3.6, 6.2) compared to prior non-responders (2.5 months, 95 % CI, 1.58, 3.42) in univariate (p = 0.064) and multivariate analysis (HR: 0.70, 95 % CI: 0.43-1.15, p = 0.156).

**Conclusion:**

Our data lends support to the concept of clonal evolution, though the clinical impact appears less robust than previously reported. Further work to determine which patients benefit from retreatment post progression is needed.

**Electronic supplementary material:**

The online version of this article (doi:10.1186/s12885-015-1701-3) contains supplementary material, which is available to authorized users.

## Background

Colorectal cancer (CRC) is one of the most common cancers worldwide. Systemic therapy is the mainstay of management for patients with metastatic CRC, involving the use of active cytotoxic drugs and biological agents either in combination or as single agents. Two antibodies targeting the epidermal growth factor receptor (EGFR), cetuximab and panitumumab, have been approved for the treatment of metastatic CRC. Activating mutations downstream to EGFR, especially in the *RAS* superfamily of oncoproteins (i.e. NRAS, KRAS) have been correlated with lack of response to anti-EGFR therapy. In 2009, the FDA restricted the use of cetuximab and panitumumab to patients lacking mutations in exon 2 (codons 12 + 13) of KRAS [1, 2].

Recently, mutations in *KRAS* have been detected in circulating tumor DNA in colorectal cancer patients with *KRAS*-wildtype (wt) cancers who had progressed on anti-EGFR therapy. Mathematical modeling of such resistance suggested that subclones harboring the *KRAS* mutations were present in low frequency in the tumor before treatment [3]. This finding supports the theory that the mechanism of resistance to anti-EGFR agents may be from intratumor heterogeneity and clonal evolution via drug-selection [4]. Based upon this theory a treatment break after developing acquired anti-EGFR resistance may allow the dominant clone that is *KRAS*-wt to repopulate and render a tumor sensitive to anti-EGFR therapy.

Success with a retreatment strategy utilizing targeted therapy has been reported with other agents in different types of cancer, such as trastuzumab in breast cancer [5] and sunitinib in gastrointestinal stromal tumor [6]. Recent small studies have suggested a benefit from a retreatment strategy in colorectal cancer with the use of anti-EGFR therapy [7–9]. In a phase II study by Santini *et al*. 39 patients with KRAS exon 2-wt metastatic CRC who had previously progressed following an initial clinical benefit to cetuximab-based therapy, were retreated with cetuximab and irinotecan. Results demonstrated an overall response rate of 53.8 %, stable disease rate was 35.9 %, and the median progression-free survival was 6.6 months [8]. Metges *et al*. (PANERB trial) prospectively treated 32 KRAS wild-type metastatic CRC patients with cetuximab and irinotecan followed by panitumumab monotherapy after progression. In 11 patients who had previously responded to cetuximab and irinotecan, an objective response rate of 22 % to panitumumab, including a disease control rate (objective response plus stable disease) of 73 % was observed [10]. In heavily pretreated patients without acquired resistance to prior cetuximab-based regimens, panitumumab obtained 67 % disease control rate and 30 % objective response rate, with median PFS of 4.2 and median OS of 9.6 months [11].

In this study, we reviewed 89 patients with advanced *KRAS* exon 2-wt CRC who had progressed on anti-EGFR therapy and were subsequently retreated on an anti-EGFR containing phase I/II clinical trial. Our goal was to evaluate the impact of both prior anti-EGFR response and interval length from prior anti-EGFR therapy upon the outcome of patients retreated with anti-EGFR therapy.

## Methods

### Patient selection

Patients with *KRAS* exon 2 (codons 12 + 13)-wt CRC who had progressed on their previous anti-EGFR-based therapy (cetuximab or panitumumab) and subsequently received at least two doses of an anti-EGFR monoclonal antibody in the context of a phase I or phase I/II clinical trial at MD Anderson Cancer Center were eligible for analysis on or before 2/27/2013. Progression on prior anti-EGFR based therapy prior to retreatment clinical trial was based upon retrospective review of the medical records. As this was a retrospective study informed consent was waived by the MD Anderson Cancer Center Institutional Review Board.

### Tissue samples and mutation analyses

All histology was centrally reviewed at MD Anderson. All tissue samples were obtained and molecularly tested as part of standard of care. Mutational results for KRAS exon 2 (codons 12 and 13) and when available extended KRAS, NRAS, BRAF V600E, and PIK3CA were recorded from standard of care mutational results done in accordance with the Clinical Laboratory Improvement Amendment (CLIA)-certified Molecular Diagnostic Laboratory within the Division of Pathology and Laboratory Medicine at MD Anderson. DNA was extracted from macro-dissected, paraffin­embedded tumor sections and over the time period studied three testing methodologies were utilized. In 85 cases PCR-based DNA sequencing for KRAS codons 12 and 13 [exon 2] with and without codon 61 [exon 3] and 146 [exon 4] was used. In 8 cases a MassARRAY platform [12] for hotspots in 11 cancer genes including KRAS codons 12 + 13 [exon 2], 61 [exon 3], and146 [exon 4], NRAS codons 12 + 13 [exon 2] and 146 [exon 4], BRAF V600E, and PIK3CA exon 9 and 20 hotspots was used. In 5 cases an Ampli-Seq 46 gene cancer panel using Ion Torrent PGM Sequencer [13] (Life Technologies, CA) including KRAS codons 12 + 13 + 19 + 22 [exon 2], 61 [exon 3], 146 [exon 4], and NRAS codons 12 + 13 + 18 [exon 2] and 61 [exon 3], BRAF V600E, and PIK3CA exon 9 and 20 hotspots was used. The lower limit of detection is 10 % for the first two methodologies and 5 % for the third. In a subset of cases additional PCR-based DNA sequencing was conducted for BRAF V600E (*n* = 54), NRAS (*n* = 12), and PIK3CA (*n* = 24).

### Data collection

Clinical information included age, race, the date of initial diagnosis and staging, KRAS, NRAS, BRAF, and PIK3CA mutational status of the tumor specimen, prior treatment history, baseline Eastern Cooperative Oncology Group (ECOG) performance status (PS), serum albumin, serum lactate dehydrogenase (LDH), and number of tumor metastatic sites were collected at the initiation of anti-EGFR retreatment. Two individual reviewers worked independently on reviewing patient electronic medical records and crosschecking the collected data.

For patients treated on more than one anti-EGFR-based regimen before retreatment (*n* = 18), data from the last anti-EGFR-based therapy were used for analysis. Response prior to the retreatment clinical trial was defined as a radiographic response or stable disease ≥6 months determined by the treating physician’s records. Responses on the retreatment clinical trial were prospectively determined for each clinical trial and categorized per RECIST 1.0 [14] or 1.1 [15] criteria. Clinical benefit on anti-EGFR retreatment clinical trial was defined as complete response (CR), partial response (PR), or stable disease (SD). Progression-free survival (PFS) was calculated as the time from the start of therapy to the first observation of disease progression or death, whichever occurred first. Patients without progression were censored on February 27, 2013.

### Statistical analysis

Univariable analyses for clinical benefit/response and PFS included chi-square and log-rank tests, respectively. Multivariable analyses for response and PFS utilized a multiple logistic regression and a Cox proportional hazards model, respectively. Covariates included in the multivariable models were response on prior anti-EGFR treatment, interval between conclusion of previous anti-EGFR treatment and initiation of anti-EGFR retreatment, age, race, gender, PS, and Royal Marsden Hospital (RMH) prognostic score comprising points for serum LDH and albumin levels, and number of metastatic sites [16]. The variables that were included in the multivariable model in the present study have been included in multivariable models in previous studies of phase 1 clinical trials [17, 18]. All of these variables were included in the multivariable model so as to avoid confounding bias that could potentially result from exclusion of specific variables [19]. All statistical analyses were carried out using SPSS 19 (SPSS Chicago, IL) by our biostatisticians GG and KH.

## Results

### Patient and treatment characteristics

We identified 97 *KRAS* exon 2-wt CRC patients who were treated on a phase I or phase I/II clinical trials containing anti-EGFR therapy and had progressed on prior cetuximab- or panitumumab-containing regimens from 5/2007 to 12/2012. An additional 8 patients (4 with NRAS mutations and 4 with BRAF V600E mutations) were excluded. The final analyzed dataset consisted of 89 patients, who were predominantly Caucasian (71 %), younger age (<60 years old, 64 %), and evenly distributed in gender, Table 1. At the initiation of the anti-EGFR re-challenge, they had good PS (ECOG < = 1, 94 %), normal albumin levels (81 %) and elevated LDH levels (79 %).

**Table 1:** Patients’ demographic and baseline clinical characteristics (*n* = 89)

Characteristic	Count (%)
Gender, *n* (%)	
Male	45 (49)
Female	44 (51)
Age, *n* (%)	
< 60 years	57 (64)
≥ 60 years	32 (36)
Race, *n* (%)	
Non-Hispanic White	63 (71)
African-American	18 (20)
Hispanic	8 (9)
Performance status, *n* (%)	
0	35 (39)
1	49 (55)
2	5 (6)
Histological grade	
Well	3 (3)
Moderate	69 (78)
Poor	17 (19)
Number of metastatic sites	
< 3	42 (47)
≥ 3	47 (53)
Serum albumin	
Normal	72 (81)
Low (<3.5 g/dL)	17 (19)
Serum LDH	
Normal	19 (21)
Elevated (>618 IU/L)	70 (79)
RMH Score	
< 2	43 (48)
≥ 2	46 (52)
*KRAS* exon 2 wild-type^a^	89 (100)
KRAS non-exon 2 mutations (*n* = 70)	0
NRAS mutations (*n* = 23)	0
BRAF V600E mutation (*n* = 64)	0
PIK3CA mutations (*n* = 37)	6

^a^KRAS exon 2 wild-type status included codons 12 and 13 (exon 2); non-exon 2 KRAS mutations included exon 3 in 55 cases and exon 3 + 4 in 15 cases; NRAS mutations included exons 2 in 23, exon 3 in 17 and exon 4 in 6; PIK3CA mutations included hotspots within exons 9 and 20

Prior anti-EGFR therapy was combined with chemotherapy in 90 % (80/89) and consisted of single agent anti-EGFR therapy in 9 % (8/89). Anti-EGFR therapy on the retreatment clinical trials utilized cetuximab in all cases. In the retreatment clinical trials, cetuximab was combined with chemotherapy and a targeted therapy in 73 % (65/89) and with a targeted therapy alone in 27 % (24/89), Table 2. Prior response, defined as response or stable disease ≥6 months, was seen in 41.6 % (37/89) of patients on prior anti-EGFR-based therapies. The median interval time between prior and retreatment anti-EGFR-based therapy was 4.6 months (range: 0.46-58.7). No additional RAS pathway mutations were identified in the 70 patients tested for KRAS exons 3 or 4, or for the 23 patients tested for NRAS. A BRAF V600E mutation was absent in the 64 tested patients and a PI3KCA mutation was identified in 6 of 37 tested patients.

**Table 2:** Characteristics related to prior and retreatment anti-EGFR regimens

Characteristic	N/total # pts (%)
Response on prior anti-EGFR therapy	
Response or stable disease ≥6 m	37/89 (42)
No response or stable disease <6 m	52/89 (58)
Clinical benefit on anti-EGFR retreatment	
Best response CR/PR/SD	50/86 (58)
Best response PD	36/86 (42)
Prior anti-EGFR-based regimens	
Panitumumab monotherapy	6/89 (7)
Panitumumab + Chemotherapy^a^	9/89 (10)
Panitumumab and AMG-102/AMG-479	1/89 (1)
Cetuximab monotherapy	2/89 (2)
Cetuximab + Chemotherapy^b^	71/89 (80)
Anti-EGFR-based retreatment regimens	
Cetuximab, FOLFOX, and dasatinib	31/89 (35)
Cetuximab, irinotecan, and bevacizumab	12/89 (13)
Cetuximab and erlotinib	13/89 (15)
Cetuximab and sirolimus	11/89 (12)
Cetuximab, HAI^c^ oxaliplatin, 5-FU, bevacizumab	20/89 (23)
Cetuximab, HAI oxaliplatin, and bevacizumab	2/89 (2)
Interval length between prior and retreatment anti-EGFR therapies	Months
Median	4.57
Mean ± Standard Deviation	7.34 ± 8.9
Range	0.46 – 58.7

^a^Chemotherapy regimen: irinotecan (7), FOLFIRI (1), 5-FU and irinotecan (1)

^b^Chemotherapy regimen: Irinotecan (42), FOLFIRI (15), FOLFOX (5), irinotecan and arq197 (3), irinotecan and apomab (1), irinotecan and bevacizumab (3), FOLFOX and dasatinib (1), Xelox (1)

^c^HAI = Hepatic Arterial Infusion

### Improved clinical benefit to cetuximab-based clinical trial retreatment in prior responders

**Fig. 1:**
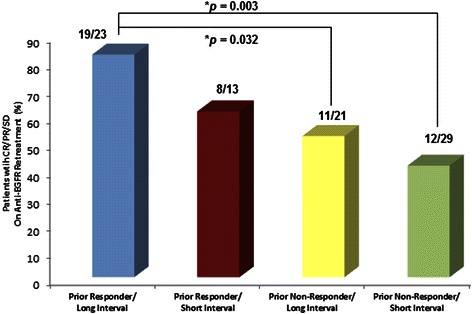
Prior responders with longer interval length were more likely to respond to anti-EGFR retreatment. Prior responders with longer interval length (longer intervening time between prior anti-EGFR therapy and anti-EGFR retreatment) were more likely to respond to anti-EGFR retreatment by analysis combining prior response to anti-EGFR retreatment and intervening time interval between anti-EGFR treatments: short (< median) or long (≥ median)

Of the 86 patients with response information on the cetuximab-based retreatments, a clinical benefit, defined as a CR, PR, or SD, occurred in 58 % (50/86) of patients. Of the 13 patients who were treated with cetuximab and erlotinib, 1 had a PR, 4 SD, and 8 PD as best response. In univariate analyses, patients who responded to the prior anti-EGFR-based regimens were more likely to obtain a clinical benefit to cetuximab-based retreatment compared to the prior non-responders (*p* = 0.007), Table 3. In addition, a trend was noted where patients with longer (≥ median) interval length between prior and retreatment anti-EGFR therapy were more likely to respond to cetuximab-based retreatment compared with patients with shorter interval length (< median), *p* = 0.053. Other factors such as race (*p* = 0.14), age (*p* = 0.99), serum albumin (*p* = 0.95), LDH (*p* = 0.28), RMH prognostic score (*p* = 1.0), PS (*p* = 0.53) or number of metastatic sites (*p* = 0.49), were not statistically associated with obtaining a response to cetuximab-based retreatment, Table 3. In addition, patients were significantly more likely to respond to cetuximab-based retreatment if they were prior responders to anti-EGFR therapy and were retreated after a longer interval than prior non-responders after either a longer (*p* = 0.032) or a shorter interval, *p* = 0.003, Fig. 1.

**Table 3:** Univariate associations between clinical benefit and PFS on anti-EGFR-based clinical trial retreatment

Patient characteristics	Clinical benefit			PFS			
	Total (*n*)	Responded *n* (%)	*p*	Total (*n*)	Progressed (*n*)	Median (95 % CI)	*p*
Response to prior anti-EGFR treatment							
No	50	23 (46)	0.007	52	45	2.50 (1.58, 3.42)	0.064
Yes	36	27 (75)		37	31	4.90 (3.60, 6.20)	
Interval length between treatments							
< median	42	20 (48)	0.053	44	40	3.20 (1.97, 4.43)	0.286
≥ median	44	30 (68)		45	36	4.10 (2.61, 5.59)	
Race/Ethnicity							
Non-White	24	17(71)	0.138	26	22	5.20 (3.94, 6.46)	0.034
White	62	33(53)		63	54	3.00 (1.92, 4.08)	
Gender							
Female	43	24 (56)	0.662	44	41	3.80 (2.88, 4.72)	0.323
Male	43	26 (61)		45	35	3.70 (2.48, 4.92)	
Age							
< 60 years	55	32 (58)	0.992	57	50	3.70 (2.91, 4.49)	0.619
≥ 60 years	31	18 (58)		32	26	3.80 (2.03, 5.57)	
PS (ECOG)							
0	32	20 (63)	0.528	35	28	3.80 (2.67, 4.93)	0.286
≥ 1	54	30 (56)		54	48	3.60 (2.08, 5.12)	
Number of metastatic sites							
< 3	42	26 (62)	0.489	42	35	4.50 (3.03, 5.97)	0.077
≥ 3	44	24 (55)		47	41	3.20 (1.92, 4.48)	
Serum albumin							
Normal	69	40 (58)	0.949	72	61	3.80 (3.18, 4.42)	0.920
Low	17	10 (59)		17	15	3.00 (0.00, 6.77)	
Serum LDH							
Normal	19	9 (47)	0.281	19	15	2.80 (0.37, 5.24)	0.667
Elevated	67	41(61)		70	61	3.80 (3.05, 4.55)	
RMH score							
0 or 1	43	25 (58)	1.000	43	35	3.70 (2.00, 5.40)	0.408
2 or 3	43	25 (58)		46	41	3.80 (2.81, 4.79)

A multiple logistic regression model confirmed that response on prior anti-EGFR therapy was a significant predictor for clinical benefit on anti-EGFR retreatment (OR: 3.38, 95 % CI: 1.27, 9.31, *p* = 0.019), Table 4. Despite the lack of statistical significance for the interaction of treatment response and interval length in the multivariate model, the markedly increased odds ratio for the combination of long interval length and prior treatment response (OR 6.7) in comparison to either long interval (OR 1.6) or prior treatment response alone (OR 2.3) suggests that such an interaction may exist but that we had insufficient data to detect it as statistically significant, Additional file 1: Table S1.

**Table 4:** Multivariate models for clinical benefit and PFS on anti-EGFR-based clinical trial retreatment

Characteristic	Clinical Benefit			PFS		
	OR	95 % CI	*p* ^a^	HR	95 % CI	*p* ^b^
Responded on prior anti-EGFR treatments, yes vs. no	3.38	(1.27, 9.31)	0.019	0.70	(0.43, 1.15)	0.156
Interval length, ≥ median vs. < median	2.37	(0.89, 6.31)	0.086	0.72	(0.45, 1.16)	0.177
Race, White vs. non-White	0.41	(0.13, 1.25)	0.116	1.75	(1.02, 3.01)	0.043
Age, ≥ 60 years vs. < 60 years	0.70	(0.25, 1.96)	0.500	1.10	(0.65, 1.87)	0.718
Gender, male vs. female	1.28	(0.50, 3.25)	0.611	0.78	(0.49, 1.24)	0.295
RMH Score, ≥ 2 vs. < 2	0.79	(0.29, 2.11)	0.633	1.32	(0.81, 2.16)	0.273
PS by ECOG, ≥ 1 versus < 1	0.76	(0.28, 2.10)	0.597	1.25	(0.76, 2.05)	0.381

^a^Based on a multivariable logistic regression model

^b^Based on a multivariable Cox proportional hazards model

*OR* Odds Ratio, *HR* Hazard Ratio

Multivariable models were tested for a possible interaction between response on prior anti-EGFR therapy vs. non-response on prior therapy (1, 0) and interval length at or above the median vs. below the median (1, 0). The interaction term between response on prior anti-EGFR therapy vs. non-response on prior therapy (1, 0) and interval length at or above the median vs. below the median (1, 0) was not significant in either model. Thus, the interaction term was not included in the final multivariable models

### PFS on cetuximab-based clinical trial retreatment were marginally increased in prior-responders

**Fig. 2:**
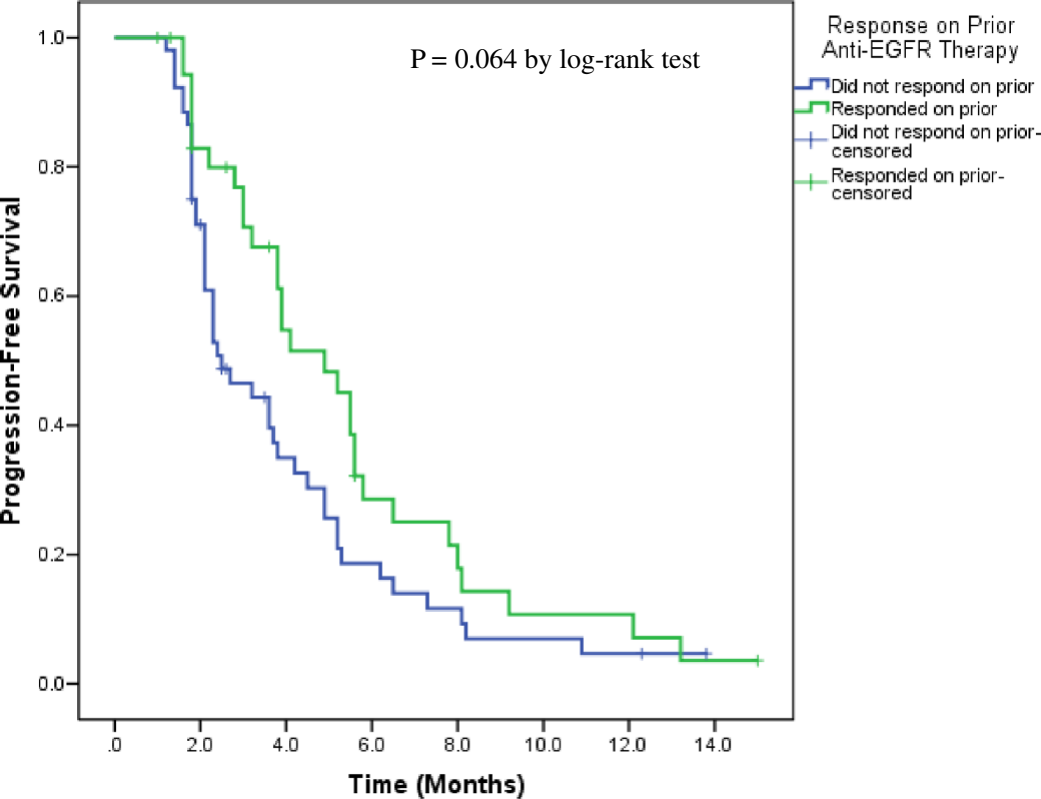
PFS on cetuximab-based retreatments by response on prior anti-EGFR-based therapies. Median PFS on the anti-EGFR-based retreatments was 4.90 months (95 % CI: 3.59, 6.20) in prior responders compared to that of 2.5 months (95 % CI, 1.58, 3.42) in prior non-responders (*p* = 0.064)

Median PFS on cetuximab-based retreatment was 4.9 months (95 % CI: 3.59, 6.20) in prior responders compared to 2.5 months (95 % CI, 1.58, 3.42) in prior non-responders, *p* = 0.064, Fig. 2. No statistically significant differences with regard to PFS were seen for the other variables, such as interval length between prior anti-EGFR-based therapy and cetuximab retreatment (*p* = 0.29), age (*p* = 0.62), number of metastatic sites (*p* = 0.07), serum albumin levels (p = 0.92), serum LDH levels (*p* = 0.67), RMH prognostic score (*p* = 0.41) or PS (*p* = 0.29) at the initiation of retreatment, Table 3. The multivariable Cox proportional hazards model revealed no significant difference in PFS on cetuximab retreatment according to prior anti-EGFR response (HR: 0.70, 95 % CI: 0.43-1.15, *p* = 0.156), interval length between prior anti-EGFR-based therapy and cetuximab retreatment (HR: 0.72, 95 % CI: 0.45-1.16, *p* = 0.177), or other demographic or clinical variables, Table 4.

## Discussion

This study shows anti-tumor activity with anti-EGFR retreatment in *KRAS* exon 2-wt CRC patients who had progressed on prior cetuximab- or panitumumab-based treatment. Prior responders were more likely to achieve a clinical benefit on the cetuximab containing retreatment clinical trial. In addition a longer interval length between prior and retreatment anti-EGFR therapy demonstrated a non-significant trend favoring an increased likelihood of obtaining a clinical benefit from anti-EGFR retreatment. This study lends support to the notion of anti-EGFR retreatment in metastatic CRC, however the magnitude of benefit from retreatment appears less than previously reported [8, 9].

In the phase II prospective study by Santini *et al.*, cetuximab retreatment resulted in an overall response rate of 53.8 %, stable disease rate was 35.9 %, and the median progression-free survival was 6.6 months [8]. The median interval time between therapies was 6 months whereas in our study it was 4.6 months. Similar to Santini *et al*. a response to prior anti-EGFR therapy was defined as a response or stable disease lasting ≥ 6 months, however our definition was based upon clinical reports and not RECIST. Another phase II prospective study by Fora *et al*. reported benefit from EGFR retreatment with a higher dose of cetuximab (500 mg/m2 weekly) in combination with irinotecan in 20 KRAS-wt metastatic CRC patients who had previously progressed on both agents [9]. A clinical benefit was seen in 9 patients (1 PR and 8 SD) and in an exploratory analysis patients treated >2 months from prior cetuximab progression had an improved PFS, *p* = 0.02). In the PANERB trial that prospectively treated 32 KRAS wild-type metastatic CRC patients with cetuximab and irinotecan followed by panitumumab monotherapy after progression, an objective response rate of 22 % to panitumumab, including a disease control rate (objective response plus stable disease) of 73 % was observed in 11 patients who had previously responded to cetuximab and irinotecan [10].

Recent data has demonstrated that mutations in NRAS exons 2, 3 and 4, and KRAS exon 3 and 4, termed extended RAS testing, confer resistance to anti-EGFR therapy in metastatic CRC. [20–22] A recent meta-analysis has estimated the prevalence of KRAS exon 3 and 4 mutations to be 11 %, and NRAS mutations to be 9.1 % [23]. It has also been reported that a more sensitive technology may detect additional mutations that confer resistance to anti-EGFR therapies [24]. A fundamental limitation of this report is that due to the use of standard of care testing, we were not able to exclude all patients with innate anti-EGFR resistance due to extended RAS mutations, as testing was done for non-exon 2 KRAS mutations in 70 patients, 79 %, and for NRAS mutations in 23 patients, 26 %.

A number of recent reports utilizing circulating free DNA have correlated the occurrence of EGFR resistance with the acquirement of mutations in both KRAS and NRAS, as well as other acquired alterations such as EGFR mutation or MET amplification [3, 25, 26]. In addition, the exact threshold for determining RAS mutational status is uncertain with data from the CRYSTAL study suggesting improved discrimination with a mutation threshold down to 5 % [27]. Within this report we are unable to address these various resistance mechanisms retrospectively as both tumor tissue and blood were inconsistently collected across the various clinical trials studied. However, these recent findings regarding acquired resistance mechanisms to anti-EGFR therapy provide clear insights into future studies to help refine and better predict which patients are truly benefiting from an anti-EGFR retreatment strategy.

This study lends supports to the concept of intratumor heterogeneity and clonal evolution via drug selection as a mechanism of resistance to anti-EGFR agents. Clonal evolution generated by genetic instability and genetic drift is not a new concept [28]. Evidence of intratumor heterogeneity and branched evolutionary growth has been revealed in solid tumors via both tissue sections and multi-region sequencing [29–32]. Kreso *et al*., followed the repopulation dynamics of 150 single lentivirus-marked lineages from ten human CRCs through serial xenograft passages in mice using DNA copy number profiling, sequencing and lentiviral lineage tracking. This study showed that individual tumor cells within a uniform genetic lineage remained stable on serial transplantation, but were functionally heterogeneous with variable chemotherapy tolerance [33]. In metastatic CRC Morelli *et al.* analyzed circulating cell free DNA and tissue samples collected from EGFR refractory patients and demonstrated that the percent of acquired KRAS mutant alleles detected in plasma declined with greater time away from anti-EGFR therapy [26]. Repopulation of sensitive subclones after the cessation of treatment has been noted in a number of model systems [34, 35]. Retreatment has demonstrated efficacy in multiple types of tumors in clinical trials [34, 36]. In non-small cell lung cancer cell lines, evolutionary mathematical modeling of tumor behavior demonstrated that optimally timed sequential strategies yielded large improvements in survival outcome with anti-EGFR treatment [37, 38].

Our study is limited by the use of heterogenous anti-EGFR retreatments. Effects of the combined regimens may obscure the differential effectiveness of the anti-EGFR retreatment. Due to the heterogeneity of included clinical trials and the sample size, the impact of each individual re-treatment regiment could not be determined. Although all anti-EGFR retreatment efficacy assessments were conducted on prospective clinical trials, the evaluations of prior anti-EGFR therapies were based on retrospective review, and due to the inability to review these scans response criteria were not based upon RECIST. In addition, the dose escalation of these phase 1 studies precluded a small subset of patients from receiving the full dose of cetuximab. As this study utilized standard of care mutation testing the majority of patients in this study did not have extended RAS testing of NRAS and non-exon 2 KRAS. In addition highly sensitive mutational testing methodology were not utilized in these patients and thus the impact of low frequency RAS mutations could not be determined [24]. Thus, our results may be confounded by the presence of a small subset of patients with innate anti-EGFR resistance from pre-existing mutations. Despite these limitations, this study represents the largest metastatic CRC anti-EGFR retreatment population published, and has attempted to evaluate both prior EGFR response and duration from prior therapy in contributing to anti-EGFR retreatment efficacy.

## Conclusions

In conclusion, this study supports the further exploration of anti-EGFR retreatment in metastatic CRC, which is ongoing with both the CRICKET (NCT02296203) and REGAIN (NCT02316496) phase II clinical trials investigating retreatment with cetuximab following prior anti-EGFR progression. Understanding the mechanisms of acquired resistance to anti-EGFR therapy in CRC will enable the improved identification of patients who are likely to benefit from a retreatment approach. However, at the present time, rechallenge with an anti-EGFR therapy remains investigational and should be conducted in the context of a clinical trial.

## Additional file

Additional file 1: Table S1. Combined effect of prior response and long interval upon clinical benefit on anti-EGFR clinical trial retreatment. (DOC 30 kb)

## References

[1] Douillard JY, Siena S, Cassidy J, Tabernero J, Burkes R, Barugel M (2014). Final results from PRIME: randomized phase III study of panitumumab with FOLFOX4 for first-line treatment of metastatic colorectal cancer. Ann Oncol.

[2] Modest DP, Stintzing S, Laubender RP, Neumann J, Jung A, Giessen C (2011). Clinical characterization of patients with metastatic colorectal cancer depending on the KRAS status. Anti-Cancer Drugs.

[3] Diaz LA, Williams RT, Wu J, Kinde I, Hecht JR, Berlin J (2012). The molecular evolution of acquired resistance to targeted EGFR blockade in colorectal cancers. Nature.

[4] Aparicio S, Caldas C (2013). The implications of clonal genome evolution for cancer medicine. N Engl J Med.

[5] Blackwell KL, Burstein HJ, Storniolo AM, Rugo HS, Sledge G, Aktan G (2012). Overall survival benefit with lapatinib in combination with trastuzumab for patients with human epidermal growth factor receptor 2-positive metastatic breast cancer: final results from the EGF104900 Study. J Clin Oncol.

[6] Bracci R, Maccaroni E, Cascinu S (2013). Transient sunitinib resistance in gastrointestinal stromal tumors. N Engl J Med.

[7] Tonini G, Imperatori M, Vincenzi B, Frezza AM, Santini D (2013). Rechallenge therapy and treatment holiday: different strategies in management of metastatic colorectal cancer. J Exp Clin Cancer Res.

[8] Santini D, Vincenzi B, Addeo R, Garufi C, Masi G, Scartozzi M (2012). Cetuximab rechallenge in metastatic colorectal cancer patients: how to come away from acquired resistance?. Ann Oncol.

[9] Fora AA, McMahon JA, Wilding G, Groman A, Ma WW, Romano KS (2013). A phase II study of high-dose cetuximab plus irinotecan in colorectal cancer patients with KRAS wild-type tumors who progressed after standard dose of cetuximab plus irinotecan. Oncology.

[10] Wadlow RC, Hezel AF, Abrams TA, Blaszkowsky LS, Fuchs CS, Kulke MH (2012). Panitumumab in patients with KRAS wild-type colorectal cancer after progression on cetuximab. Oncologist.

[11] Pietrantonio F, Perrone F, Biondani P, Maggi C, Lampis A, Bertan C (2013). Single agent panitumumab in KRAS wild-type metastatic colorectal cancer patients following cetuximab-based regimens: Clinical outcome and biomarkers of efficacy. Cancer Biol Ther.

[12] Gabriel S, Ziaugra L, Tabbaa D (2009). SNP genotyping using the Sequenom MassARRAY iPLEX platform. Curr Protoc Hum Genet.

[13] Singh RR, Patel KP, Routbort MJ, Reddy NG, Barkoh BA, Handal B (2013). Clinical validation of a next-generation sequencing screen for mutational hotspots in 46 cancer-related genes. J Mol Diagn.

[14] Choi H, Charnsangavej C, Faria SC, Macapinlac HA, Burgess MA, Patel SR (2007). Correlation of Computed Tomography and Positron Emission Tomography in Patients With Metastatic Gastrointestinal Stromal Tumor Treated at a Single Institution With Imatinib Mesylate: Proposal of New Computed Tomography Response Criteria. J Clin Oncol.

[15] Eisenhauer EA, Therasse P, Bogaerts J, Schwartz LH, Sargent D, Ford R (2009). New response evaluation criteria in solid tumours: revised RECIST guideline (version 1.1). Eur J Cancer.

[16] Wheler J, Tsimberidou AM, Hong D, Naing A, Falchook G, Piha-Paul S (2012). Survival of 1,181 patients in a phase I clinic: the MD Anderson Clinical Center for targeted therapy experience. Clin Cancer Res.

[17] Garrido-Laguna I, Janku F, Vaklavas C, Falchook GS, Fu S, Hong DS (2012). Validation of the Royal Marsden Hospital prognostic score in patients treated in the Phase I Clinical Trials Program at the MD Anderson Cancer Center. Cancer.

[18] Hong DS, Patel JC, Wheler J, Naing A, Garrido-Laguna I, Falchook G (2012). Outcomes in 144 patients with colorectal cancer treated in a phase I clinic: the MD Anderson Cancer Center experience. Clin Colorectal Cancer.

[19] MH K: Multivariable Analysis: A Practical Guide for Clinicians and Public Health Researchers, 3rd edn. Cmabridge CB2 8BS, United Kingdom: Cambridge University Press; 2011

[20] Douillard JY, Oliner KS, Siena S, Tabernero J, Burkes R, Barugel M (2013). Panitumumab-FOLFOX4 treatment and RAS mutations in colorectal cancer. N Engl J Med.

[21] Schwartzberg LS, Rivera F, Karthaus M, Fasola G, Canon JL, Hecht JR (2014). PEAK: A Randomized, Multicenter Phase II Study of Panitumumab Plus Modified Fluorouracil, Leucovorin, and Oxaliplatin (mFOLFOX6) or Bevacizumab Plus mFOLFOX6 in Patients With Previously Untreated, Unresectable, Wild-Type KRAS Exon 2 Metastatic Colorectal Cancer. J Clin Oncol.

[22] Heinemann V, von Weikersthal LF, Decker T, Kiani A, Vehling-Kaiser U, Al-Batran SE (2014). FOLFIRI plus cetuximab versus FOLFIRI plus bevacizumab as first-line treatment for patients with metastatic colorectal cancer (FIRE-3): a randomised, open-label, phase 3 trial. Lancet Oncol.

[23] Sorich MJ, Wiese MD, Rowland A, Kichenadasse G, McKinnon RA, Karapetis CS: Extended RAS mutations and anti-EGFR monoclonal antibody survival benefit in metastatic colorectal cancer: a meta-analysis of randomized controlled trials. Ann Oncol 2014, doi:10.1093/annonc/mdu378 10.1093/annonc/mdu378 25115304

[24] Molinari F, Felicioni L, Buscarino M, De Dosso S, Buttitta F, Malatesta S (2011). Increased detection sensitivity for KRAS mutations enhances the prediction of anti-EGFR monoclonal antibody resistance in metastatic colorectal cancer. Clin Cancer Res.

[25] Misale S, Yaeger R, Hobor S, Scala E, Janakiraman M, Liska D (2012). Emergence of KRAS mutations and acquired resistance to anti-EGFR therapy in colorectal cancer. Nature.

[26] Morelli MP, Overman MJ, Dasari A, Kazmi SMA, Vilar Sanchez E, Eng C (2013). Heterogeneity of acquired KRAS and EGFR mutations in colorectal cancer patients treated with anti-EGFR monoclonal antibodies. ASCO Meeting Abstracts.

[27] Van Cutsem E, Lenz H-J, Kohne CH, Heinemann V, Tejpar S, Melezinek I (2015). Fluorouracil, leucovorin, and irinotecan plus cetuximab treatment and RAS mutations in colorectal cancer. J Clin Oncol.

[28] Nowell PC (1976). The clonal evolution of tumor cell populations. Science.

[29] Cook HC (1997). Origins of … tinctorial methods in histology. J Clin Pathol.

[30] Gerlinger M, Rowan AJ, Horswell S, Larkin J, Endesfelder D, Gronroos E (2012). Intratumor heterogeneity and branched evolution revealed by multiregion sequencing. N Engl J Med.

[31] Khalique L, Ayhan A, Weale ME, Jacobs IJ, Ramus SJ, Gayther SA (2007). Genetic intra-tumour heterogeneity in epithelial ovarian cancer and its implications for molecular diagnosis of tumours. J Pathol.

[32] Campbell PJ, Yachida S, Mudie LJ, Stephens PJ, Pleasance ED, Stebbings LA (2010). The patterns and dynamics of genomic instability in metastatic pancreatic cancer. Nature.

[33] Kreso A, O'Brien CA, van Galen P, Gan OI, Notta F, Brown AM (2013). Variable clonal repopulation dynamics influence chemotherapy response in colorectal cancer. Science.

[34] Kuczynski EA, Sargent DJ, Grothey A, Kerbel RS (2013). Drug rechallenge and treatment beyond progression--implications for drug resistance. Nat Rev Clin Oncol.

[35] Crockford A, Jamal-Hanjani M, Hicks J, Swanton C (2014). Implications of intratumour heterogeneity for treatment stratification. J Pathol.

[36] Naing A, Agarwal R, Falchook G, Hong DS, Janku F, Wheler J (2013). Retreatment after secondary resistance or mixed response: a pilot study. Oncology.

[37] Chmielecki J, Foo J, Oxnard GR, Hutchinson K, Ohashi K, Somwar R (2011). Optimization of dosing for EGFR-mutant non-small cell lung cancer with evolutionary cancer modeling. Sci Transl Med.

[38] Mumenthaler SM, Foo J, Leder K, Choi NC, Agus DB, Pao W (2011). Evolutionary modeling of combination treatment strategies to overcome resistance to tyrosine kinase inhibitors in non-small cell lung cancer. Mol Pharm.

