# Supplementary material for: Retreatment with anti-EGFR based therapies in metastatic colorectal cancer: impact of intervening time interval and prior anti-EGFR response
Source: BMC Cancer. 2015 Oct 16;15:713. doi: 10.1186/s12885-015-1701-3 (PMC4609167; doi:10.1186/s12885-015-1701-3)
Supplement: Additional file 1: Table S1. — Combined effect of prior response and long interval upon clinical benefit on anti-EGFR clinical trial retreatment. (DOC 30 kb) [file 12885_2015_1701_MOESM1_ESM.doc]

**Supplementary Material Available Online**

**Table S1:** Combined effect of prior response and long interval upon clinical benefit on anti-EGFR clinical trial retreatment

| Interval | Prior Response | N | Clinical Benefit (%) | Odds Ratio | 95% Confidence Interval | *P* |
| --- | --- | --- | --- | --- | --- | --- |
| Short | No | 29 | 12 (41%) | 1.0 | - | - |
| Long | Yes | 23 | 19 (83%) | 6.7 | 1.8-25 | 0.004 |
| Short | Yes | 13 | 8 (62%) | 2.3 | 0.6-8.7 | 0.231 |
| Long | No | 21 | 11 (52%) | 1.6 | 0.5-4.8 | 0.442 |
